# Sweroside Alleviated Aconitine-Induced Cardiac Toxicity in H9c2 Cardiomyoblast Cell Line

**DOI:** 10.3389/fphar.2018.01138

**Published:** 2018-10-25

**Authors:** Li-Qun Ma, You Yu, Hui Chen, Mei Li, Awais Ihsan, Hai-Ying Tong, Xian-Ju Huang, Yue Gao

**Affiliations:** ^1^College of Life Sciences, South-Central University for Nationalities, Wuhan, China; ^2^College of Pharmacy, South-Central University for Nationalities, Wuhan, China; ^3^Department of Biosciences, COMSATS University Islamabad (CUI), Sahiwal, Pakistan; ^4^School of Traditional Chinese Medicine, Beijing University of Chinese Medicine, Beijing, China; ^5^National Demonstration Center for Experimental Ethnopharmacology Education, South-Central University for Nationalities, Wuhan, China; ^6^Department of Pharmacology and Toxicology, Beijing Institute of Radiation Medicine, Beijing, China

**Keywords:** aconitine, sweroside, cardiac toxicity, calcium overload, reactive oxygen species

## Abstract

Aconitine is the main bioactive ingredient of Aconitum plants, which are well-known botanical herbs in China. Aconitine is also notorious for its high cardiotoxicity, as it can induce life-threatening ventricular arrhythmias. Unfortunately, there are few effective antidotes to aconitine toxicity. This study aimed to evaluate the potent protective effects of the ingredients from *V. baillonii* on aconitine toxicity on H9c2 cell line. Cell viability was assessed by methylthiazoltetrazolium bromide (MTT). Intracellular Ca^2+^ concentration alteration and reactive oxygen species (ROS) generation were observed by confocal microscopy and flow cytometry, respectively. Cellular oxidative stress was analyzed by measuring malondialdehyde (MDA) and superoxide dismutase (SOD) levels. Mitochondrial membrane potential (ΔΨ) was determined using JC-1 kit. RT-PCR and Hoechst staining techniques were conducted to determine the levels of autophagy/apoptosis. The mRNA levels of dihydropyridine receptor (DHPR), ryanodine receptors (RyR2) and sarcoplasmic reticulum Ca^2+^-ATPase (SERCA) were measured by RT-PCR. We screened six components from *V. baillonii*, among which, sweroside exhibited the strongest protective effects on aconitine-induced cardiac toxicity. Sweroside suppressed the aconitine-induced mRNA expressions of Na_V_1.5 (encoded by SCN5A), RyR2 and DHPR, and reversed the aconitine-induced decrease in mRNA level of SERCA, thus preventing the aconitine-induced persistent intracellular Ca^2+^ accumulation and avoiding intracellular Ca^2+^ overload. We further found that sweroside restabilized the aconitine-disrupted mitochondrial membrane potential (ΔΨ) and reversed the aconitine-induced increase in the mRNA levels of cell autophagy-related factors (Beclin-1, Caspase-3, and LC3- II) in H9c2 cells. In the whole-animal experiments, we observed that sweroside (50 mg/kg) alleviated effectively aconitine-induced arrhythmias by analysis of electrocardiogram (ECG) recording in rats. Our results demonstrate that sweroside may protect cardiomyocytes from aconitine toxicity by maintaining intracellular Ca^2+^ homeostasis, restabilizing mitochondrial membrane potential (ΔΨ) and avoiding cell autophagy/apoptosis.

## Introduction

Aconitum plants, have been extensively used for centuries as traditional herbal medicines for a wide range of human maladies including rheumatism, inflammation in oriental countries. Aconitine is the main pharmacological ingredient in Aconitum plants. Despite the therapeutic benefits, aconitine is one of known cardiotoxins and neurotoxins (Honerjager and Meissner, 1983; Catterall et al., 1992; Ameri, 1998; Chan, 2009), it can cause lethal malignant ventricular arrhythmias. Aconitine and its analogs have even been developed into the experimental tools to establish cardiac arrhythmic models in modern heart research (Herzog et al., 1964; Sato et al., 1979; Hikino et al., 1980; Fraser et al., 2003). In clinic, herb-induced aconitine poisoning occur from time to time, as the narrow therapeutic index (Lin et al., 2004). However, the detoxification strategies for aconitine are still limited to supportive treatment, such as gastric lavage, charcoal hemoperfusion, catheterization, injection of atropine, or magnesium sulfate, and so on (Lin et al., 2004; Gottignies et al., 2009; Clara et al., 2015). Although, amiodarone was reported to successfully treat the aconitine-induced life-threatening arrhythmias, lidocaine and other classic antiarrhythmic drugs failed (Yeih et al., 2000), the paucity of effective and specific agents for aconitine poisoning is obvious. The aconitine toxicity, thus, needs a more detailed investigation to develop more effective and safe antidotes.

**FIGURE 1:**
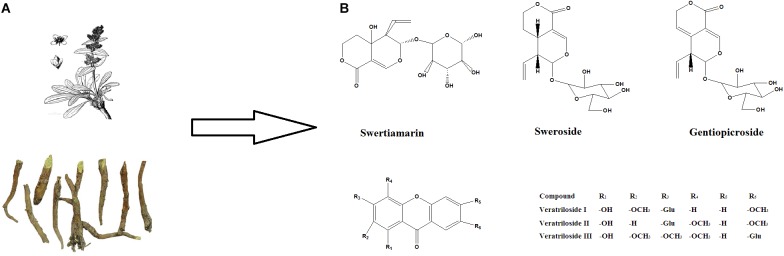
The main ingredients in *V. baillonii.* and their chemical structures. **(A)** The dried stems and roots of *V. baillonii*. **(B)** The chemical structures of swertiamarin, sweroside, and gentiopicroside, which are the main activate components in *V. baillonii*.

*Veratrilla baillonii* Franch (*V. baillonii*) belongs to the family of Gentianaceae (Figure 1A). It is an ethnodrug that has long been used in the Southwest of China (Yang and Zhou, 1980). Local clinical evidence has indicated that *V. baillonii* possesses multiple pharmacological potential, as diverse as antipyretic, anti-inflammation and curing gastrospasm. Local people also believe that *V. baillonii* has preventative effects on toxicity from aconitum plants (Yang and Zhou, 1980). Our previous studies further supported this belief, as we observed that aconitine *in vivo* damaged the heart, liver, kidney and brain in rat, but the water extract of *V. baillonii*, which contains abundant iridoids (Huang et al., 2016), could attenuate the pathological changes of both acute (Ge et al., 2016) and subacute (Yu et al., 2016) toxicities induced by *Aconitum brachypodum* Diels (*A. brachypodum*). However, whether this protective action is mediated by these iridoids and the clear molecular mechanism remain to be elucidated.

Here, in this study, we evaluated the detoxication effects of active components from *V. baillonii* on aconitine-induced H9c2 cell injury, and the mechanism was explored in details. Three iridoids, gentiopicroside, and sweroside, swertiamarin, and three veratrilosides I, II, and III, were selected to evaluate their influences on cell activity by MTT assay and by measuring the lactate LDH release. The most promising active chemical was then determined. Flow cytometry and real-time PCR were used to further elucidate the mediation of autophagy, intracellular calcium ions, intracellular ROS, and oxidative stress induction. Whole animal experiments were performed to test whether the compounds possess potential anti-arrhythmia effects in aconitine-induced arrhythmia rat model.

We found that sweroside, one of the iridoids from *V. baillonii*, exhibited protective effects on cardiomyocytes against aconitine toxicity, and maintaining intracellular Ca^2+^ homeostasis and avoiding cell autophagy/apoptosis may be involved in this protection.

## Materials and Methods

### Guideline Statement

All methods used in this study are in accordance with protocols approved by the South-Central University for Nationalities. All animal studies comply with the Guide for the Ethical Committee in South-Central University for Nationalities (number of authorization: SYXK20 08-0 0 05).

### Materials

Aconitine was purchased from Sigma-Aldrich (Sigma-Aldrich, St. Louis, MO, United States). Secoiridoids of gentiopicroside, sweroside and swertiamarin were obtained from Yuanye Bio-Technology Co, Ltd (Shanghai, China). Veratriloside I, II, and III were extracted by our laboratory (Huang et al., 2016). The purities of the above substances were >98% as determined by HPLC-UV. All the compounds were dissolved in DMSO and then diluted in Dulbecco’s modified Eagle’s medium (DMEM, Hyclone, Logan, UT, United States) to the desired concentrations. The final concentration of DMSO was 0.1% (v/v) (Figure 1B).

Cell culture reagent of high glucose Dulbecco’s modified Eagle’s medium (DMEM) was purchased from Hyclone (Logan, United States). Trypsin was purchased from Biosharp (Anhui, China), and fetal bovine serum (FBS) was provided by Sijiqing Co. Ltd. (Hangzhou, China). The detection kit of 2′, 7′- dichloro-dihydro-fluorescein diacetate (DCFH-DA) was purchased from Sigma–Aldrich (St. Louis, MO, United States). The cell-permeable calcium-sensitive fluorescent dye Fluo-3/AM was purchased from Beyotime Institute of Biotechnology (Shanghai, China). Fluro-4/AM was purchased from Invitrogen (Camarillo, CA, United States).

### Cell Cultures and Treatment

H9c2 cells, the rat cardiomyoblast cell line were obtained from Chinese Academy of Sciences Cell Bank (Shanghai, China). The cells were cultured in DMEM supplemented with 10% FBS, penicillin (100 U/mL) and streptomycin (100 μg/mL) in a 5% CO_2_ incubator at 37°C. The cell suspension (1 × 10^5^ cells/ml) was plated into 96 well-plates and subcultured to 60∼75% confluence for the following experiments. Different concentrations of the sample were prepared in serial dilutions, and DMSO (0.1%) was used as the control. H9c2 cells were underwent following treatments: (1) 0.01∼50 μM aconitine for 24 h, and then 10 μM aconitine for the subsequent experiments; (2) 0∼50 μM iridoidglucosides and 10 μM aconitine; (3) 0∼50 μM Xanthones for 2 h, and then 10 μM aconitine exposure for 24 h.

### Assessment of Cell Viability

Cell survival was observed using a phase-contrast microscope (OLYMPUS, Japan), and the cell viability was evaluated by the reduction of MTT and LDH release. Briefly, H9c2 cells (1 × 10^5^ cells/ml) were treated with the suggested concentrations of the chemicals for the indicated time at 37°C. After 4 h of incubation with MTT (0.5 mg/ml), the cells were lysed in DMSO and the amount of MTT formazan was qualified by determining the absorbance at 570 nm using a microplate reader (Thermo Fisher 1510, United States). The cell viability was expressed as a percent of the control culture value. The level of LDH was detected with a commercially available kit according to the manufacturer’s instruction (Jiancheng Bioengineering Institute, Nangjing, China). The absorbance was measured at 450 nm with a microplate reader as described above. LDH release (% of total) was calculated as the percentage of LDH in the medium vs. the total LDH activity in the cells.

### Measurement of MDA Production and SOD Activity

The MDA contents and SOD activity were determined using commercial kits (Nanjing Jiancheng Bioengineering Institute, Nanjing, China) according to the manufacturer’s instruction. To calculate the concentration of MDA and SOD, the protein concentrations of lysis buffer were measured by an enhanced bicinchoninic acid (BCA) protein assay kit (Beyotime Institute of Biotechnology, Shanghai, China).

### Morphological Assessment of Apoptosis

Apoptosis was determined by incubating the cells with 10 μg/mL Hoechst 33258 for 30 min at 37°C using the Hoechst staining kit (Beyotime Institute of Biotechnology, Shanghai, China) according to the manufacturer’s protocol. Nuclear morphological changes in the cells were observed under an Olympus fluorescence microscope (Tokyo, Japan) at a 461 nm emission. The apoptotic cells appeared with strong blue fluorescence, while the normal cells appeared with only weak fluorescence.

### Measurement of Mitochondrial Membrane Potential (ΔΨ)

The change in the mitochondrial membrane potential (ΔΨ) was determined using the JC-1 kit (Beyotime Institute of Biotechnology, Shanghai, China). H9c2 cells from different treatment groups were washed with DMEM and incubated with JC-1 (1 μM) in DMEM at 37°C for 20min. After washing with DMEM, the cells were immediately detected by flow cytometry. The ratio of aggregated and monomeric JC-1 was used to quantify the change of ΔΨ. A decreased JC-1 ratio represents the depolarization of the mitochondria, indicating a decrease in ΔΨ.

### Flow Cytometry for the Analysis of Intracellular ROS Generation

Dichloro-dihydro-fluorescein diacetate fluorescent probe was employed to monitor intracellular ROS. Cells in logarithmic growth phase were incubated in 6-well plates for 24 h for stabilization, then the medium was replaced with those containing 10 μmol/L aconitine or aconitine (10 μmol/L)+sweroside (2–20 μM) for 24 h to observe the effects. After exposure, the cells were washed with fresh DMEM three times, and then they were resuspended at a concentration of 1 × 10^6^ cells/ml and incubated with 10 μM DCFH-DA at 37°C for 30 min before being detected and analyzed by flow cytometry. A minimum of 20,000 events were analyzed per sample and the results were expressed as the fold-change of the fluorescence intensity over the control.

### Measurement of Intracellular Ca^2+^ Levels by Flow Cytometry and Confocal Microscopy

Employing Ca^2+^ indicators, Fluo-4/AM and Fluo-3/AM, to measure the intracellular Ca^2+^ concentrations ([Ca^2+^]_i_). Briefly, the cells in logarithmic growth phase were seeded in 6-well plates and incubated for 24 h for stabilization, then the medium was replaced with those containing 10 μmol/L aconitine or aconitine (10 μmol/L)+sweroside (2–20 μM) and incubated for 24 h to observe the effects. After exposure, the cells were washed with fresh DMEM three times; then, the H9c2 cells were loaded with 5 μmol/L Fluo-3/AM for 30 min at 37°C in dark. The fluorescence intensity was analyzed by flow cytometry.

In confocal experiments, H9c2 cells were seeded in circular discs and incubated with 10 μM sweroside for 2 h, then loaded with 2 μM Fluo-4/AM (dissolved in 0.1% DMSO plus pluronic acid (Life Technologies, Carlsbad, CA, United States) for 15 min at 37°C in Tyrode’s solution containing (in mmol/L): NaCl 143, KCl 5.4, CaCl_2_ 1.8, Na_2_H_2_PO_4_ 0.3, MgCl_2_ 0.5, glucose 5.5, and HEPES 5, pH7.4 adjusted with 10M NaOH. The intracellular Ca^2+^ concentration was evaluated by measuring the fluorescence intensity excited at 488 nm and emitted at 510 nm using the LSM 700 Laser Confocal Microscopy System (Carl Zeiss, Berlin, Germany). The fluorescence was read every 5 s, and aconitine was administered into the discs at a fixed time point. The fluorescence intensity was determined before and after administrating drugs.

### RNA Extraction and Real-Time Polymerase Chain Reaction (RT-PCR)

Real-time PCR was employed determine the gene expression. Briefly, the total RNA was extracted from H9c2 cells with the Trizol reagent (Qiagen, Valencia, CA United States). For mRNA quantification, cDNA was synthesized from 1 μg of total RNA using the PrimeScript^TM^ RT reagent kit (TaKaRa, Dalian, China) following the manufacturer’s instructions. Reactions were performed in a 20 μL volume according to the thermal cycler manufacturer’s protocol (Rox) using an ABI 7500 Real-Time PCR system (Applied Biosystems) under the condition: 30 s at 95°C, followed by 40 cycles of 15 s at 95°C, and 34 s at 60°C (Ge et al., 2016). The primer sequences used for the amplification of target genes are listed in Table 1.

**Table 1:** Primer sequences used for the amplification of target genes.

Gene	Forward	Reverse
GAPDH	TGTGTCCGTCGTGGATCTGA	TTGCTGTTGAAGTCGCAGGAG
Beclin-1	GCCTCTGAAACTGGACACG	CCTCTTCCTCCTGGCTCTCT
Caspase-3	GGAGCAGTTTTGTGTGTGTGA	AGTTTCGGCTTTCCAGTCAG
LC-3	ACCCTCTACGATGCTGGTGA	GCTGTCCTCAATGTCCTTCTG
DHPR	CATCTTTGGATCCTTTTTCGTTCT	TCCTCGAGCTTTGGCTTTCTC
RyR2	TGCTGCGAGCCGGG	TGGCGGTGGCGTAGGA
SERCA2a	CAGCCATGGAGAACGCTCA	TCGTTGACCCCGAAGTGG
SCN5A	CACCCTCAACCTCTTCATCG	CTTCTTCTGCTCCTCCGTCA


### Animals and Administration of Drugs

Male Sprague-Dawley rats (200–250 g) were supplied by the Center of Laboratory Animals of Hubei Province (Wuhan, China).

A total of 30 rats were used in these serials of experiments. SD rats were randomly divided into three groups (6 for the control group, 12 for aconitine group, 12 for sweroside + aconitine group). The rats in control group received no treatment. The rats in sweroside + aconitine group received intraperitoneal injection (i.p.) of sweroside (50 mg/kg) for 5 days, once per day. Water and food were freely available for all of the animals.

### ECG Recording

Pentobarbital sodium (3% dissolved in physiological salt; 90 mg/kg) was used for anesthesia. Standard Lead II ECG recording was carried out with a polygraph recorder (ADInstruments Pty Ltd, Australia). After a 15 min stabilization period, vehicle (i.e., physiological salt, i.p.) was administered to the control group, and aconitine (1 mg/kg, i.p.) were administered to induce arrhythmias in both aconitine group and sweroside + aconitine group. When the arrhythmias activity lasted and the ECG recording was continuously conducted 90 min post-administration. Data analysis was performed by using ECG Auto analysis software, Labchart Pro 8.

### Statistical Analysis

All statistical analyses were performed in Origin 9.0 software (Origin Lab, Northampton, MA, United States). Data are presented as mean ± standard deviation (SD) and were analyzed with one-way analysis of variance (ANOVA) or Student-Newman-Keuls test. *P* < 0.05 was considered statistically significant.

## Results

### Sweroside Displayed Potential Protective Efficiency on Aconitine-Induced Cytotoxicity in H9c2 Cells

**FIGURE 2:**
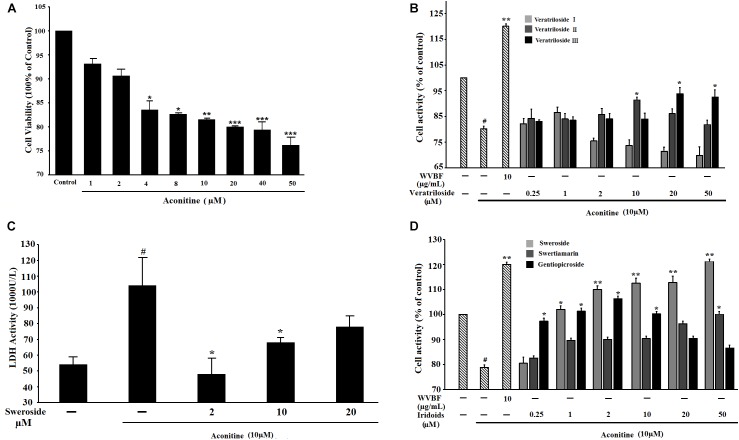
The influence of active components from *V. baillonii* on aconitine-induced cytotoxicity in H9c2 cells. **(A)** The cell viability influenced by aconitine for 24 h; **(B)** The cell viability influenced by pretreatment with iridoidglucosides for 2 h, then exposure to aconitine for 24 h; **(C)** Effect of sweroside on LDH level in H9c2 cells subjected to aconitine; **(D)** The cell viability influenced by pretreatment with xanthones for 2 h, then exposure to aconitine for 24 h. *n* = 3 (*n*, the number of experiments), #*P* < 0.05 vs. control, ^∗^*P* < 0.05, ^∗∗^*P* < 0.01, and ^∗∗∗^*P* < 0.001 vs. aconitine group. WVBF: water decoction of *Veratrilla baillonii*.

We first calculated the cytotoxicity of aconitine. As shown in Figure 2A, aconitine (4–50 μM) dose-dependently reduced the cell viability of H9c2 cells after incubation for 24 h. The IC_50_ value was 32 μM. Then, we screened six compounds and found that each of them could improve the cell survival, which had been reduced by 10 μM aconitine (Figure 2B) to varying degrees. Among these compounds, Veratrilosides I, II, and III showed weaker protection on H9c2 cells than WVBF (10 μg/ml) or secoiridoids. It was sweroside that exhibited the greatest efficiency among them (Figure 2D), so in the subsequent experiments, we explored its protective activity and the underlying mechanism in detail. 2 μM sweroside initiated the significant increase in the cell viability (*P* < 0.01 vs. aconitine group), and the efficiency reached its maximum at a concentration of 50 μM, a level similar to that of WVBF (10 μg/ml). Since LDH is widely used as a marker of cellular damage, the cell injury was assessed by determining LDH activity. The LDH leakage increased markedly in the aconitine group compared with the control group, but this increase was significantly blocked by sweroside treatment (2–10 μM) in a dose-dependent manner (Figure 2C). Together, these findings indicated that sweroside could promote cell survival and reduce cell damage in H9c2 cells subjected to aconitine.

### Sweroside Pretreatment Reduced Aconitine-Induced Oxidative Stress and Intercellular ROS Production in H9c2 Cells

**FIGURE 3:**
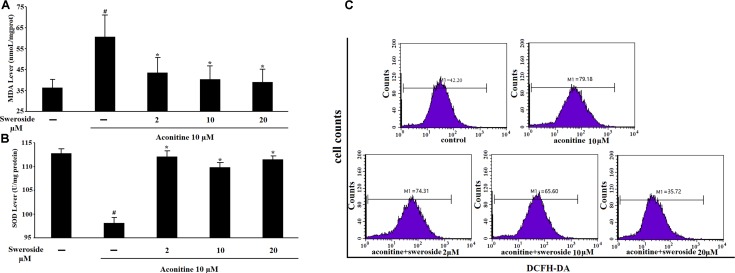
The influence of sweroside on aconitine-induced oxidative stress and intercellular ROS production in H9c2 cells. **(A)** Effects of sweroside on MDA content under aconitine treatment in H9c2 cells; **(B)** Effects of sweroside pretreatment on SOD product in H9c2; **(C)** Flow cytometry analysis of aconitine-induced ROS. *n* = 3 (*n*, the number of experiments). ^#^*P* < 0.05 vs. control, ^∗^*P* < 0.05 vs. aconitine group.

Oxidative stress is one of the direct drivers of cell damage, it is an increase in the cellular and tissue concentrations of reactive oxygen species (ROS) due to peroxidation. We tested the level of lipid peroxidation by measuring the MDA level, which is the end product of lipid peroxidation. As illustrated in Figure 3A, the exposure of the cells to 10 μM aconitine resulted in a significant increase of the MDA level (*P* < 0.05) compared to that of control cells. This increase in the content of MDA induced by aconitine was blocked significantly by pretreatment the cells with sweroside (2, 10, 20 μM) (*P* < 0.05) (Figure 3A).

The effect of sweroside on the intracellular ROS production induced by aconitine was shown in Figure 3C. The cells incubated with aconitine produced stronger DCF signals than the control group. However, incubation with both aconitine and a set of concentrations of sweroside (2–20 μM) resulted in a marked reduction in the DCF fluorescence (Figure 3C), indicating an inhibitory effect of sweroside on aconitine-induced intracellular ROS production.

The SOD activity was measured to further confirm the antioxidative ability of sweroside. As illuminated in Figure 3B, aconitine remarkably reduced the SOD activity, however, pre-incubation with sweroside significantly reversed this reduction (*P* < 0.05 vs. aconitine group).

### Sweroside Inhibited the Increases in the mRNA Levels of Cell Autophagy/Apoptosis-Related Factors Induced by Aconitine in H9c2 Cells

**FIGURE 4:**
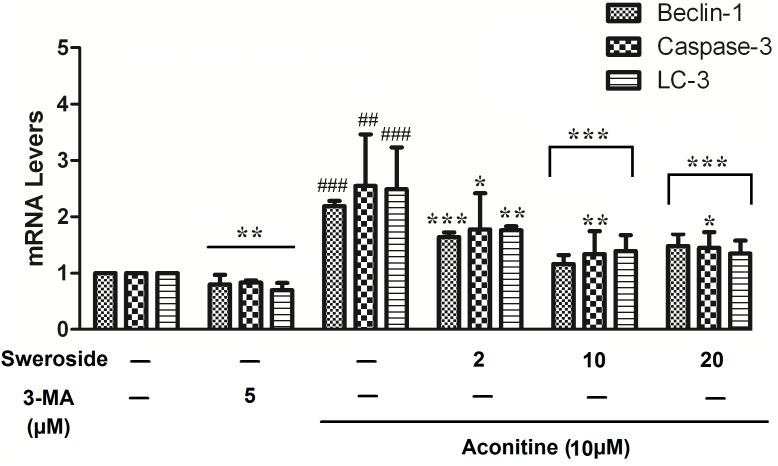
Effects of sweroside pretreatment on cell autophagy/apoptosis-related gene mRNA expression levels in H9c2 cells. *n* = 3 (*n*, the number of experiments). ^#^*P* < 0.05, ^##^*P* < 0.01, and ^###^*P* < 0.001 vs. control, ^∗^*P* < 0.05, ^∗∗^*P* < 0.01, and ^∗∗∗^*P* < 0.001 vs. aconitine group.

To further study the protective effects of sweroside on aconitine-induced cardiac toxicity, a classical autophagy inhibitor, 3-methyladenine (3-MA), which is a phosphoinositide 3-kinase inhibitor that exerts its autophagy-inhibited effect before the formation of the autophagosome was used as a pharmacological tool. LC3- II is a useful indicator of autophagosome initiation, and the LC3-II levels can rise with autophagy. As shown in Figure 4, aconitine could significantly increase the LC3- II and Beclin-1 mRNA levels (*P* < 0.01 vs. control group), indicating the inductive potential of autophagy; it could also lead to a marked increase of cleaved Caspase-3 mRNA levels as indicated (*P* < 0.01 vs. control group), suggesting its inductive potential for apoptosis. However, treatment with sweroside could substantially down-regulate all of these increased indexes induced by aconitine, which is a similar result to that of the positive control 3-MA. These results suggest that sweroside could protect cardiomyocytes via suppressing cell autophagy/apoptosis induced by aconitine.

### Sweroside Prevented Aconitine-Induced Apoptosis Through Mitochondrion-Dependent Apoptotic Pathway

**FIGURE 5:**
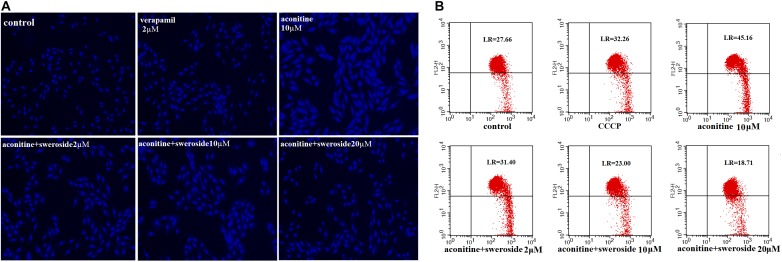
The effect of Sweroside on aconitine-induced apoptosis and mitochondrial membrane potential changes. **(A)** Apoptosis was detected by staining with Hoechst 33258; **(B)** Flow cytometry analysis of aconitine-induced mitochondrial injury.

Normal living cells with Hoechst staining showed uniform blue fluorescence in the control group, while apoptotic cells showed hyperchromatic and dense fluorescent particles within the massive apoptotic nuclear cytoplasm by fluorescence microscopy in the aconitine group. As shown in Figure 5A, sweroside could attenuate the cell apoptosis induced by aconitine.

The ultrastructural changes in H9c2 cells were examined to evaluate the myocardial damage induced by aconitine application. The control vs. sweroside group showed no significant difference. However, the proportion of the JC-1 monomer drastically increased in the group with aconitine application compared to the control; this increase was significantly blunted by sweroside pretreatment (Figure 5B). Mitochondrion is the key organelle mainly responsible for cell energy supply and cellular apoptosis. A decrease in the ΔΨ causes membrane depolarization and triggers a cascade of apoptotic signaling (Hamacher-Brady et al., 2007). In the present study, JC-1 staining showed that sweroside pretreatment abated the aconitine-induced depolarization of the membrane potential in H9c2 cells (Figure 5B).

### Sweroside Partially Blunted the Intracellular Ca^2+^ Increase Induced by Aconitine

**FIGURE 6:**
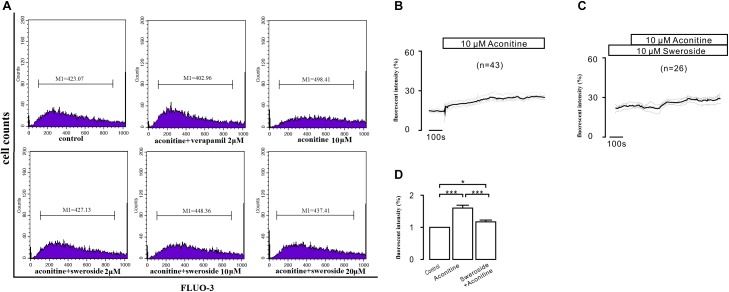
The influence of sweroside on the intracellular Ca^2+^ increase induced by aconitine. **(A)** The effects of calcium overload were analyzed by flow cytometry using the fluorescent probe Fluo-3/AM; **(B,C)** Changes in the intracellular calcium ion levels in H9c2 cells treated with different drug Hank’s media; **(D)** Effects of pretreatment with sweroside and aconitine-stimulation on [Ca^2+^]_i_ in H9c2 cells. ^∗^*P* < 0.05 and ^∗∗∗^*P* < 0.001 compared between two groups.

As shown in Figure 6A, 10 μM aconitine significantly potentiated Fluo-3 fluorescence, demonstrating an elevation in intracellular Ca^2+^ concentration. Sweroside (2–10 μM) drastically inhibited this elevation of the intracellular Ca^2+^.

To further validated the result above, we performed experiments to continually observe the dynamic changes of [Ca^2+^]_i._ The administration of 10 μM aconitine led to a persistent and stable increase in [Ca^2+^]_i_ compared with that before the administration (*P* < 0.001), as shown in Figure 6B. However, pretreatment with sweroside for 2 h eliminated this aconitine-induced [Ca^2+^] elevation (Figure 6C). The fluorescence intensity changes coincide with the above description (Figure 6D).

### Sweroside Reversed the Aconitine-Induced Changes of the mRNA Levels of DHPR, RyR2, and SERCA2a

**FIGURE 7:**
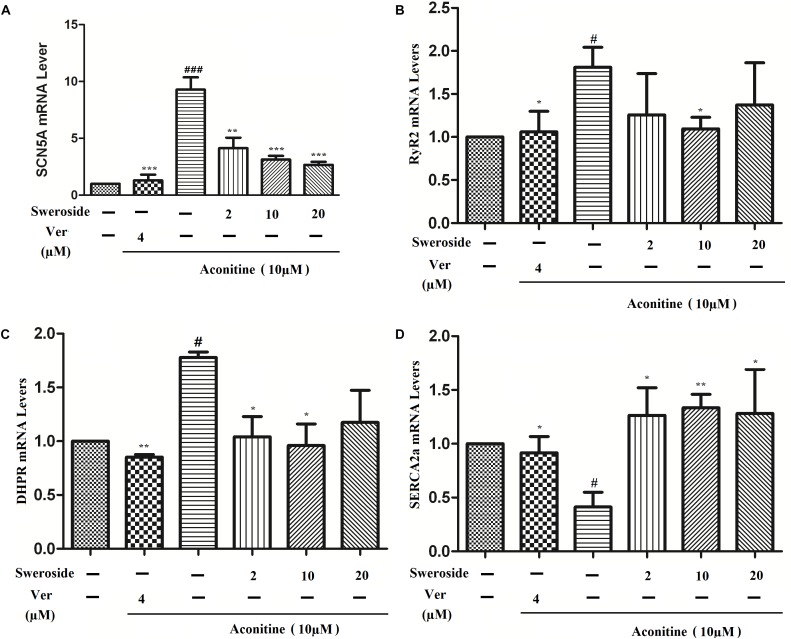
Effects of sweroside pretreatment on calcium-related gene mRNA expression in H9c2 cells. **(A)** SCN5A mRNA expression level was measured; the data showed that aconitine markedly increased SCN5A mRNA expression, however, sweroside effectively reversed this increase. **(B–D)** Sweroside reduced aconitine-induced enhancement of RyR2, DHPR, SERCA2a mRNA expression, respectively. *n* = 3 (*n*, the number of experiments), ^#^*P* < 0.05 and ^##^*P* < 0.01 vs. control, ^∗^*P* < 0.05, ^∗∗^*P* < 0.01, and ^∗∗∗^*P* < 0.001 vs. aconitine group.

The total RNA of the H9c2 cells was isolated and analyzed by semi-quantitative RT-PCR. Cells exposed to such dose showed significant differences between the aconitine group and the sweroside treatment group (Figure 7). The increased mRNA expression levels of DHPR, SCN5A and RyR2 in the aconitine group (*P* < 0.05) suggested that aconitine could up-regulate L-type voltage-dependent Ca^2^^+^ channels (LVDCCs), sarcoplasmic reticulum Ca^2+^ release channels and Na^+^ channels. Aconitine exposure could moderately suppress SERCA2a mRNA level (*P* < 0.05), indicating that aconitine inhibited the activity of SERCA2a. The increased mRNA expression level of DHPR and RyR2 induced by aconitine were reversed almost completely by pretreatment with verapamil and sweroside, respectively, compared to the aconitine group (*P* < 0.05). Both verapamil and sweroside also noticeably recovered the aconitine-induced suppression of the SERCA2a expressions noticeably (*P* < 0.01), suggesting that sweroside acts against the aconitine-induced stimulation on the cardiomyocytes ion channels.

### Sweroside Ameliorated Aconitine-Induced Arrhythmias in Rats

**FIGURE 8:**
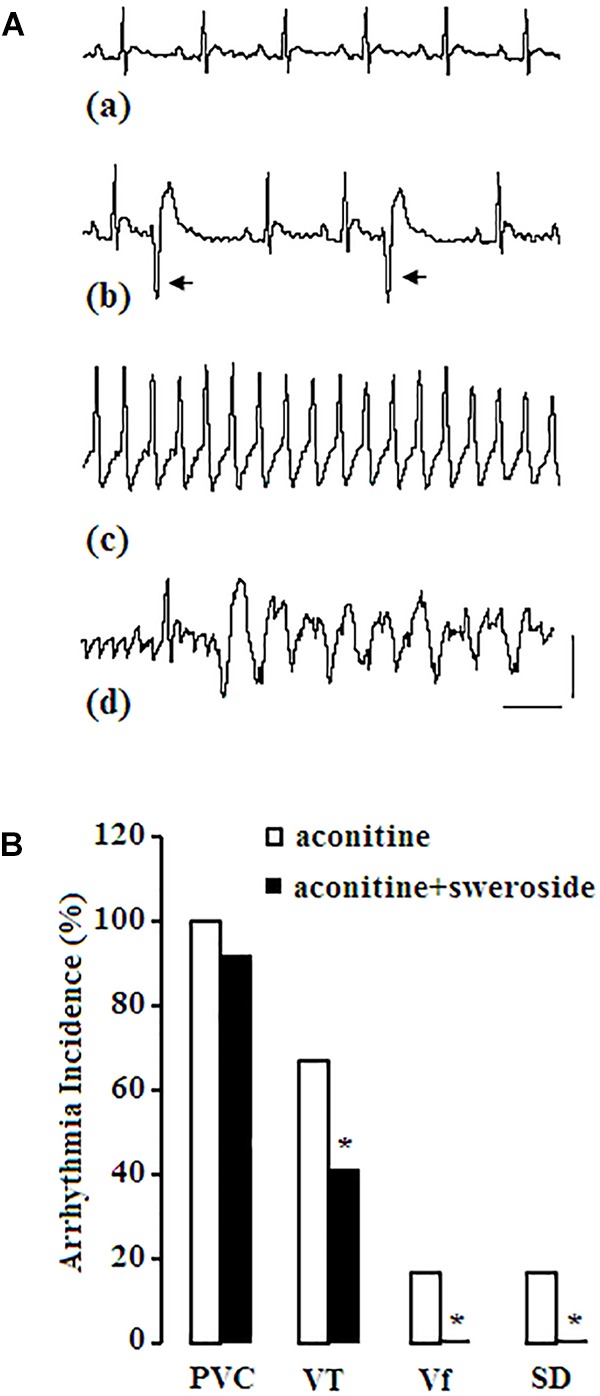
Effect of sweroside on aconitine-induced arrhythmia activities in rats. (A) aconitine induced various types of arrhythmias. Panel (a), normal sinus rhythm (NSR) before administration of aconitine; (b), aconitine induced proarrhythmic premature ventricular complex (PVC); (c), aconitine induced ventricular tachycardia (VT); (d), aconitine induced ventricular fibrillation (Vf). Arrow indicates PVC; horizontal line indicates 150 s, vertical line indicates 1mv; (B) sweroside reduced the incidence of VT, Vf, and sudden death (SD) induced by aconitine. ^∗^*P* < 0.05 vs. aconitine group.

Finally, we assessed whether prevention of sweroside on H9c2 cells could ameliorate cardiac arrhythmias induced by aconitine in rats. To test this hypothesis, we performed whole rat experiments. As shown in Figure 8A, in aconitine group, all of the rats were observed to occur proarrhythmic premature ventricular complex (PVC) during the ECG recording, 66.7% of rats developed into VT, 16.7% of which even rapidly deteriorated into Vf, subsequently, died. In sweroside treatment group, before aconitine administration, the ECG recording had no changes, compared with the control group (data not shown); after aconitine administration, the majority of rats occurred arrhythmias during the ECG recording, however, there was a significantly reduced incidence of VT, Vf, and sudden death (SD) compared with aconitine group (Figure 8B). Thus, sweroside ameliorated aconitine-induced ventricular arrhythmias and reduced the risk of death. In control group, there was no abnormality observed in ECG recording.

## Discussion

Although the cardiac toxicity of aconitine has been widely recognized (Honerjager and Meissner, 1983; Catterall et al., 1992; Ameri, 1998), there are few antidotes available to countact the deadly symptoms induced by aconitine or aconitum plants. In this study, we observed that aconitine caused severe cell damage in H9c2 cell line, and sweroside strongly prevented the cells from such damage. These findings are consistent with our previous *in vivo* data (Yu et al., 2016). The aconitine-induced toxicity in H9c2 cells is characterized by increased oxidative stress, inducing autophagy/apoptosis, disturbing mitochondrion the potential and triggering Ca^2+^ overload.

Secoiridoids, a type of monoterpenes that has the general form of cyclopentanopyran with one of the rings open, are the characteristic components of the medicinal plants of the Gentianaceae family, which includes gentiopicroside, sweroside and swertiamarin (Herzog et al., 1964; Hikino et al., 1980; Honerjager and Meissner, 1983). The present results revealed that sweroside could remarkably attenuate the cell death/apoptosis of H9c2 cells induced by aconitine, suggesting that secoiridoids, especially sweroside, are the material basis of the therapeutic uses of *V. baillonii* for aconitine-induced toxicity. Other Gentianaceae plants containing secoiridoids may also demenstrate potent protective effect against the cardiac toxicity induced by aconitum medicine. However, the specific weight and the influences of the various secoiridoids remain unknown, which require further experimental confirmation in the future.

Calcium is one of the most crucial intracellular messengers, it functions to translate extracellular stimuli into intracellular signaling pathways that ultimately regulate cellular development, survival, differentiation and gene expression (Naranjo and Mellstrom, 2012). Therefore the intracellular calcium concentration must be precisely regulated to avoid cellular injury. This delicate calcium balance is maintained by Ca^2+^-permeable channels (mainly including DHPR and RyR2), calcium pumps, and exchangers (Berridge et al., 2000; Carafoli, 2002; Naranjo and Mellstrom, 2012). Any factors or stimuli that disrupt this well-defined Ca^2+^ balance give rise to heart disease, including arrhythmia (Greiser and Schotten, 2013). This mechanism may be an important cause for the increased arrhythmias known to occur with aconitine toxicity. Aconitine increases the accumulation of intracellular Na^+^ by up-regulation of the function of voltage-gated sodium channels, and inhibits SERCA2 expressions on the sarcoplasmic reticulum, leading to membrane depolarization (Sawanobori et al., 1987; Wright, 2002; Zhou et al., 2013). Such depolarization is expected to open DHPRs, resulting in Ca^2+^ influx into the cells, which then activates the RyR2 channels, leading to massive release of Ca^2+^ from the sarcoplasmic reticulums, and even Ca^2+^ overload. In addition, the accumulation of intracellular Na^+^ also inhibits the SERCA2 to keep cytosolic [Ca^2+^]_i_ in sustainable elevation (Li et al., 2012), thereby exacerbating Ca^2+^ overload. The most novel finding in our study is that pretreatment with sweroside reversed the aconitine-induced changes of the mRNA transcription of SCN5A, RyR2, DHPR, and SERCA2a, thereby recovering the aberrant alterations of the intracellular Ca^2+^ signals induced by aconitine.

**FIGURE 9:**
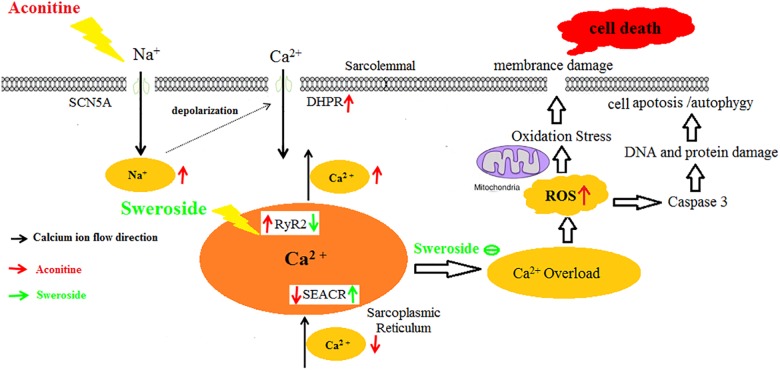
The cardiac protective mechanism of sweroside on aconitine-exposed H9c2 cells. Aconitine upregulated the function of Na^+^ channels to cause increase of intracellular Na^+^ ions leading to intracellular Ca^2+^ overload, which induced excess ROS production. The large accumulation of ROS triggered cellular autophagy/apoptosis, even death. Sweroside reversed effectively aconitine-induced cell damage.

The intracellular calcium overload could enhance intracellular ROS production, which is a well-known driver of cell apoptosis/autophagy or even cell death via the mitochondrial and caspase 3 pathways (Green and Leeuwenburgh, 2002). In this study, we observed that aconitine induced abundant ROS production in H9c2 cells. The augmented ROS directly attacks SERCA2 to decrease its activity, increasing the open probability of RyRs (Xu et al., 1998; Sun et al., 2008) to give rise to [Ca^2+^]_i_ elevation in damaged cells (Di et al., 2016), and then causing arrhythmias by apoptosis (Frey et al., 2000; Gordan et al., 2016; Llano-Diez et al., 2016). Apparently, additional ROS generation and calcium overload promote reciprocally each other, establishing a vicious circle to exacerbate cell damage. The present study revealed that sweroside could break this circle, namely, it simultaneously counteracted aconitine-induced intracellular ROS production and Ca^2+^ elevation, thus protecting the cardiocytes. The cardiacprotective mechanism of sweroside on aconitine-induced cell damage was proposed in Figure 9.

Autophagy is a dynamic process that turns over organelles and proteins through a lysosome-associated degradation process, and it serves a critical function in cellular homoeostasis by regulating cell survival and cell death pathways (Bhandary et al., 2012). Autophagy may promote cell death through excessive self-digestion and the degradation of essential cellular constituents or it may interact with the apoptotic cascade in the heart (Cao et al., 2009). Many damaged cardiomyocytes show features of autophagic/lysosomal cell death during myocardial injury, including the appearance of cytoplasmic autophagic vacuoles and the recruitment of LC3 to the autophagosomes (Hamacher-Brady et al., 2007). The present study confirmed that aconitine induced autophagy and apoptosis in H9c2 cells by significant increasing LC3- II, cleaved caspase-3 and Beclin-1 expression as well as by decreasing the ΔΨ. Sweroside, however, could protect H9c2 cells against aconitine-induced autophagy and apoptosis.

In view of the excellent protective effects of sweroside on H9c2 cells, it may be postulated that sweroside could reduce aconitine evoked arrhythmias. Whole rat experiments revealed that sweroside prevented aconitine-induced arrhythmias from deteriorating into lethal types.

In conclusion, the present study revealed that sweroside from *V. baillonii* could ameliorate the aconitine-induced cardiac toxicity *in vitro*, and reduce the arrhythmia incidence induced by aconitine *in vivo*. These results provide a valuable therapy option for the well-known toxin, aconitine. More investigations on the clinical influence of sweroside as well as its mother plants are being conducted in our lab in the future.

## Author Contributions

L-QM, YY, and X-JH designed the experiments. L-QM, YY, HC, ML, AI, H-YT, X-JH, and YG performed the experiments and analyzed the data. L-QM, YY, and X-JH wrote the paper.

## Conflict of Interest Statement

The authors declare that the research was conducted in the absence of any commercial or financial relationships that could be construed as a potential conflict of interest.

## Abbreviations

ECG: electrocardiogram
DHPR: dihydropyridine receptor
DMSO: dimethyl sulfoxide
LDH: lactate dehydrogenase
MDA: malondialdehyde
MTT: methylthiazoltetrazolium bromide
PVC: premature ventricular complex
ROS: reactive oxygen species
RyR2: ryanodine receptor
SERCA: sarco/endoplasmic reticulum Ca^2+^-ATPase
SOD: superoxide dismutase
*V. baillonii*: *Veratrilla baillonii* Franch
Vf: ventricular fibrillation
VT: ventricular tachycardia
WVBF: water decoction of *Veratrilla baillonii*

## References

[B1] Ameri A. (1998). The effects of Aconitum alkaloids on the central nervous system. *Prog. Neurobiol.* 56 211–235. 10.1016/S0301-0082(98)00037-9 9760702

[B2] Berridge M. J. Lipp P. Bootman M. D. (2000). The versatility and universality of calcium signalling. *Nat. Rev. Mol. Cell Biol.* 1 11–21. 10.1038/35036035 11413485

[B3] Bhandary B. Marahatta A. Kim H. R. Chae H. J. (2012). An involvement of oxidative stress in endoplasmic reticulum stress and its associated diseases. *Int. J. Mol. Sci.* 14 434–456. 10.3390/ijms14010434 23263672 PMC3565273

[B4] Cao D. J. Gillette T. G. Hill J. A. (2009). Cardiomyocyte autophagy: remodeling, repairing, and reconstructing the heart. *Curr. Hypertens. Rep.* 11 406–411. 10.1007/s11906-009-0070-1 19895751 PMC3005716

[B5] Carafoli E. (2002). Calcium signaling: a tale for all seasons. *Proc. Natl. Acad. Sci. U.S.A.* 99 1115–1122. 10.1073/pnas.032427999 11830654 PMC122154

[B6] Catterall W. A. Trainer V. Baden D. G. (1992). Molecular properties of the sodium channel: a receptor for multiple neurotoxins. *Bull. Soc. Pathol. Exot.* 85(5 Pt 2), 481–485. 1340350

[B7] Chan T. Y. (2009). Aconite poisoning. *Clin. Toxicol.* 47 279–285. 10.1080/15563650902904407 19514874

[B8] Clara A. Rauch S. Uberbacher C. A. Felgenhauer N. Druge G. (2015). High-dose magnesium sulfate in the treatment of aconite poisoning. *Anaesthesist* 64 381–384. 10.1007/s00101-015-0013-y 25812545

[B9] Di A. Mehta D. Malik A. B. (2016). ROS-activated calcium signaling mechanisms regulating endothelial barrier function. *Cell Calcium* 60 163–171. 10.1016/j.ceca.2016.02.002 26905827 PMC4988951

[B10] Fraser S. P. Salvador V. Manning E. A. Mizal J. Altun S. Raza M. (2003). Contribution of functional voltage-gated Na+ channel expression to cell behaviors involved in the metastatic cascade in rat prostate cancer: I. lateral motility. *J. Cell. Physiol.* 195 479–487. 10.1002/jcp.10312 12704658

[B11] Frey N. McKinsey T. A. Olson E. N. (2000). Decoding calcium signals involved in cardiac growth and function. *Nat. Med.* 6 1221–1227. 10.1038/81321 11062532

[B12] Ge Y. B. Jiang Y. Zhou H. Zheng M. Li J. Huang X. J. (2016). Antitoxic effect of *Veratrilla baillonii* on the acute toxicity in mice induced by *Aconitum brachypodum*, one of the genus Aconitum. *J. Ethnopharmacol.* 179 27–37. 10.1016/j.jep.2015.12.030 26719282

[B13] Gordan R. Fefelova N. Gwathmey J. K. Xie L. H. (2016). Involvement of mitochondrial permeability transition pore (mPTP) in cardiac arrhythmias: evidence from cyclophilin D knockout mice. *Cell Calcium* 60 363–372. 10.1016/j.ceca.2016.09.001 27616659 PMC5127715

[B14] Gottignies P. El Hor T. Tameze J. K. Lusinga A. B. Devriendt J. Lheureux P. (2009). Successful treatment of monkshood (aconite napel) poisoning with magnesium sulfate. *Am. J. Emerg. Med.* 27 755.e1–755.e4. 10.1016/j.ajem.2008.10.008 19751645

[B15] Green P. S. Leeuwenburgh C. (2002). Mitochondrial dysfunction is an early indicator of doxorubicin-induced apoptosis. *Biochim. Biophys. Acta* 1588 94–101. 10.1016/S0925-4439(02)00144-8 12379319

[B16] Greiser M. Schotten U. (2013). Dynamic remodeling of intracellular Ca(2)(+) signaling during atrial fibrillation. *J. Mol. Cell. Cardiol.* 58 134–142. 10.1016/j.yjmcc.2012.12.020 23298712

[B17] Hamacher-Brady A. Brady N. R. Logue S. E. Sayen M. R. Jinno M. Kirshenbaum L. A. (2007). Response to myocardial ischemia/reperfusion injury involves Bnip3 and autophagy. *Cell Death Differ.* 14 146–157. 10.1038/sj.cdd.4401936 16645637

[B18] Herzog W. H. Feibel R. M. Bryant S. H. (1964). The effect of aconitine on the giant axon of the squid. *J. Gen. Physiol.* 47 719–733. 10.1085/jgp.47.4.719 14127608 PMC2195349

[B19] Hikino H. Konno C. Takata H. Yamada Y. Yamada C. Ohizumi Y. (1980). Antiinflammatory principles of Aconitum roots. *J. Pharmacobiodyn.* 3 514–525. 10.1248/bpb1978.3.514 7205533

[B20] Honerjager P. Meissner A. (1983). The positive inotropic effect of aconitine. *Naunyn Schmiedebergs Arch. Pharmacol.* 322 49–58. 6302523 10.1007/BF00649352

[B21] Huang X. J. Li J. Mei Z. Y. Chen G. (2016). Gentiopicroside and sweroside from *Veratrilla baillonii* Franch. induce phosphorylation of Akt and suppress Pck1 expression in hepatoma cells. *Biochem. Cell Biol.* 94 270–278. 10.1139/bcb-2015-0173 27248905

[B22] Li L. Louch W. E. Niederer S. A. Aronsen J. M. Christensen G. Sejersted O. M. (2012). Sodium accumulation in SERCA knockout-induced heart failure. *Biophys. J.* 102 2039–2048. 10.1016/j.bpj.2012.03.045 22824267 PMC3341551

[B23] Lin C. C. Chan T. Y. Deng J. F. (2004). Clinical features and management of herb-induced aconitine poisoning. *Ann. Emerg. Med.* 43 574–579. 10.1016/j.annemergmed.2003.10.046 15111916

[B24] Llano-Diez M. Cheng A. J. Jonsson W. Ivarsson N. Westerblad H. Sun V. (2016). Impaired Ca(2+) release contributes to muscle weakness in a rat model of critical illness myopathy. *Crit. Care* 20:254. 10.1186/s13054-016-1417-z 27510990 PMC5050561

[B25] Naranjo J. R. Mellstrom B. (2012). Ca2+-dependent transcriptional control of Ca2+ homeostasis. *J. Biol. Chem.* 287 31674–31680. 10.1074/jbc.R112.384982 22822058 PMC3442502

[B26] Sato H. Yamada C. Konno C. Ohizumi Y. Endo K. Hikino H. (1979). Pharmacological actions of aconitine alkaloids. *Tohoku J. Exp. Med.* 128 175–187. 10.1620/tjem.128.175 462476

[B27] Sawanobori T. Hirano Y. Hiraoka M. (1987). Aconitine-induced delayed afterdepolarization in frog atrium and guinea pig papillary muscles in the presence of low concentrations of Ca2+. *Jpn. J. Physiol.* 37 59–79. 10.2170/jjphysiol.37.59 3497297

[B28] Sun J. Yamaguchi N. Xu L. Eu J. P. Stamler J. S. Meissner G. (2008). Regulation of the cardiac muscle ryanodine receptor by O(2) tension and S-nitrosoglutathione. *Biochemistry* 47 13985–13990. 10.1021/bi8012627 19053230 PMC2636679

[B29] Wright S. N. (2002). Comparison of aconitine-modified human heart (hH1) and rat skeletal (mu1) muscle Na+ channels: an important role for external Na+ ions. *J. Physiol.* 538(Pt 3), 759–771. 11826163 10.1113/jphysiol.2001.012915 PMC2290112

[B30] Xu L. Eu J. P. Meissner G. Stamler J. S. (1998). Activation of the cardiac calcium release channel (ryanodine receptor) by poly-S-nitrosylation. *Science* 279 234–237. 10.1126/science.279.5348.234 9422697

[B31] Yang Y. B. Zhou J. (1980). [Studies on the xanthones of *Veratrilla baillonii* Franch. I. Structures of veratriloside and veratrilogenin (author’s transl)]. *Yao Xue Xue Bao* 15 625–629. 7257783

[B32] Yeih D. F. Chiang F. T. Huang S. K. (2000). Successful treatment of aconitine induced life threatening ventricular tachyarrhythmia with amiodarone. *Heart* 84:E8. 10.1136/heart.84.4.e8 10995426 PMC1729459

[B33] Yu Y. Yi X. J. Mei Z. Y. Li J. Huang X. J. Yang G. Z. (2016). The water extract of *Veratrilla baillonii* could attenuate the subacute toxicity induced by *Aconitum brachypodum*. *Phytomedicine* 23 1591–1598. 10.1016/j.phymed.2016.10.001 27823623

[B34] Zhou Y. H. Piao X. M. Liu X. Liang H. H. Wang L. M. Xiong X. H. (2013). Arrhythmogenesis toxicity of aconitine is related to intracellular ca(2+) signals. *Int. J. Med. Sci.* 10 1242–1249. 10.7150/ijms.6541 23935402 PMC3739024

